# Contemporary Challenges in Venous Thromboembolism: Evolving Populations and Implications for Management and Risk Stratification

**DOI:** 10.3390/jcm15041509

**Published:** 2026-02-14

**Authors:** Patrick Leung, Prahlad Ho, Hui Yin Lim

**Affiliations:** 1 Department of Haematology, Northern Pathology Victoria, Northern Health, Melbourne, VIC 3076, Australia; prahlad.ho@nh.org.au; 2 Northern Clinical Diagnostics and Thrombovascular Research (NECTAR) Centre, Northern Health, Epping, VIC 3076, Australia; 3 Australian Centre for Blood Diseases, Monash University, Melbourne, VIC 3000, Australia; 4 Department of Medicine, University of Melbourne, Melbourne, VIC 3000, Australia; 5 School of Health and Biomedical Sciences, RMIT University, Bundoora, VIC 3082, Australia

**Keywords:** venous thromboembolism, epidemiology, risk factors, anticoagulants, thrombectomy, risk assessment, biomarkers

## Abstract

Venous thromboembolism (VTE) remains a major cause of morbidity and mortality globally. The incidence of VTE continues to increase over time, contributed to by demographic shifts and emerging risk factors, such as novel cancer treatments and exposure to gender-affirming hormonal therapies. While the introduction of direct oral anticoagulants (DOACs) revolutionized VTE management, increasing complexity in select patient cohorts has driven the need for alternative treatment strategies, including pharmacological and interventional approaches. Concurrently, contemporary patient populations have exposed limitations in existing risk assessment models, highlighting the need for revision and consideration of novel biomarkers. In this review, we provide an overview of the changing VTE landscape, highlighting emerging risk factors, advances in treatment, and the utility of current risk stratification tools and novel biomarkers in guiding care.

## 1. Introduction

Venous thromboembolism (VTE), which includes deep vein thrombosis (DVT) and pulmonary embolism (PE), represents a significant disease burden globally, affecting nearly 10 million individuals annually [1]. VTE not only imposes significant morbidity and mortality risk on patients but also substantial economic burden on healthcare systems, driven by hospitalisation and hospital acquired events [2,3]. The incidence of VTE only continues to increase over time. This trend has been commonly attributed to demographic shifts with an ageing population, rising prevalence of traditional risk factors such as obesity and malignancy and heightened diagnostic vigilance [4,5,6]. Emerging risk factors, including new cancer therapies [7,8] and a growing transgender population on hormonal therapy [9], have further contributed to the evolving landscape of VTE risk.

The growing burden of VTE has driven the continuous advancement in treatment strategies. Direct oral anticoagulants (DOACs) revolutionized the treatment of VTE at their introduction, providing an option that was non-inferior, and with lower bleeding risk compared to vitamin K antagonists [10,11,12,13,14]. However, with increasingly complex patient populations, there remains residual bleeding risk even with DOAC therapy which warrants optimisation, particularly given long term usage. DOACs also have important limitations in patients with chronic kidney disease (CKD) with restricted use at more advanced stages of renal impairment as well as limited availability of readily reversible agents. These limitations have led to an ongoing search for alternate treatment strategies. Factors XI (FXI) and XII (FXII) have emerged as new targets with the potential to dissociate bleeding risk from anticoagulant efficacy [15,16]. Additionally, the development of interventional strategies for treatment of VTE has provided additional options for select patient groups [17,18]. Care pathways are also evolving alongside our treatment options, allowing for the management of patients in an outpatient setting [19].

Despite the progress made in the management of VTE, risk prognostication has not advanced in parallel. Many risk assessment models rely heavily on clinical criteria or have limited incorporation of biomarkers [20,21,22]. Furthermore, these tools were developed in historical cohorts and may not reflect contemporary VTE patients, who are older, have more co-morbidities, and are increasingly managed with DOACs rather than warfarin, or are on newer therapies unaccounted for in the models, underlining the need to revise existing models and develop new tools. Current tools also have limited ability to capture dynamic risk, often relying on baseline variables. These limitations highlight the need to update existing models to incorporate contemporary treatments, more diverse patient populations and novel biomarkers, potentially supported by machine learning approaches to improve individualised risk prediction in VTE.

In this narrative review, we will explore the evolving VTE epidemiology, emerging therapies and advances in risk stratification, while highlighting future directions in management.

## 2. Methodology

A comprehensive narrative literature search was conducted to identify studies for VTE epidemiology, therapeutic strategies and risk stratification. Electronic databases (including PubMed and Embase) and grey literature sources such as Google Scholar were searched up to 29 January 2026. Supplementary Table S1 shows the database search strategy. Boolean operators were used to combine concepts, and results were restricted to human studies published in English. Reference lists of relevant articles were screened to identify additional studies. Selected studies were representative of the overall literature and chosen to illustrate key concepts and trends.

## 3. Epidemiology—Trends and Emerging Risks

### 3.1. Rising VTE Incidence

VTE incidence increases with age, with rates markedly higher in those 70 years and older [4,23]. Worryingly, the global population continues to age, with a projected doubling of those aged 65 years or older within the next 30 years [24,25]. Incidence also differs with sex, with women having a slightly higher lifetime risk compared to men; however, this varies with age, with higher incidence observed in women under 45 years and over 80 years old [23,26]. However, VTE incidence appears to be increasing over time, independent of age and sex. The Tromsø study of 27,000 Norwegians demonstrated a 27% increase in VTE over 16 years when adjusted for age [6]. The Worcester VTE study revealed similar findings, with a 25.5% increase between 2001–2009, independent of age and sex [27]. These findings highlight that a substantial proportion of VTE risk is driven by acquired and potentially modifiable factors. Notably, the rise in VTE has occurred concomitantly with an increasingly multimorbid global population [28,29,30]. As such, patients are not only increased risk of VTE, but may also carry comorbidities that limit and complicate treatment.

### 3.2. Cancer

Cancer is a key comorbidity that has been associated with the rising burden of VTE globally. In a large cohort study of almost 4 million patients in the United States, the incidence of cancer was demonstrated to be increasing over successive generations [31]. Similar patterns have been demonstrated at a global level, with an overall rise in case numbers across 200 countries between 1990–2021 [32]. Analyses of the Cancer Observatory database predict a 77% increase in cancer cases between 2022–2050, with a projected 12% increase in young adult and adolescent cases [33,34]. Consistent with the rising cancer incidence, cancer-associated thrombosis (CAT) has also increased. In a review of Danish medical records, the 12-month incidence of VTE following cancer diagnosis significantly increased from 1.0% in 1997 to 3.4% in 2017 [35]. Similar findings were highlighted in analysis of the California Cancer Registry, where the cumulative incidence of VTE rose across a range of malignancies between 2014–2017 compared to 2005–2007 [36]. Both studies concluded this increase in CAT was a combination of improved survival rates, in addition to more intensive surveillance, with Mulder et al. observing a 10-fold increase in computed tomography (CT) scans [35].

In addition to cancer itself, cancer therapies have also been associated with increased VTE risk. Longstanding treatments, such as immunomodulatory agents for multiple myeloma and hormonal therapies for breast cancer have previously been associated with increased VTE risk [37,38]. Newer therapies have also been implicated as independent risk factors for VTE. Immune checkpoint inhibitors (ICIs) are now widely incorporated into treatment regimens for a number of solid organ malignancies. While immune-related adverse events are known complications, emerging evidence suggests that these agents may also increase VTE risk. In a recent retrospective cohort study of over 10,000 patients with cancer receiving ICI [7], the 1-year cumulative incidence of VTE was 11.1%. The authors noted that VTE risk varied with ICI class, independent of cancer type or stage. An earlier study by Roopkumar et al. found a similar 1-year cumulative incidence of nearly 11% in ICI treated patients [39].

Chimeric Antigen Receptor T-cell (CAR-T) therapy is another recent advancement in cancer treatment. Used in a number of B-cell lymphomas, B-cell acute lymphoblastic leukemia and multiple myeloma, emerging evidence suggests a possible link to VTE. A meta-analysis conducted on 47 studies with a total of 7040 patients identified that thrombotic risk appeared highest in the first 6 months following CAR-T infusion (2.4% vs. 0.1% after 6 months), independent of CAR-T target, underlying malignancy or rates of cytokine release syndrome [40]. Conversely, a review of nine Phase 2 and Phase 3 CAR-T studies failed to demonstrate an associated VTE risk, with only four VTE events reported across these studies [41]. As novel cancer therapies become more commonplace, consideration and stratification of these therapies’ contribution to CAT risk will become increasingly important.

### 3.3. Obesity

Between 1990 and 2021, the prevalence of elevated body mass index (BMI) has increased by over 50% in both men and women and is projected to continue rising [42]. Elevated BMI is a well described risk factor for VTE. This association may be mediated by increased levels of tissue factor (TF) and plasminogen activator inhibitor-1 (PAI-1), leading to coagulation cascade activation and reduced fibrinolysis, promoting a prothrombotic state [43,44,45]. In a meta-analysis of six studies investigating the influence of BMI on VTE risk, obesity (BMI > 30 kg/m^2^) was associated with an odds ratio (OR) of 2.00 (95% CI 1.72–2.32), though there was significant heterogeneity between studies [46]. In another study of the Tromsø population, weight gain ≥ 7.5 kg was associated with increased VTE risk (HR 1.93; 95% CI 1.38–2.68), which was further amplified by baseline obesity (HR 3.75; 95% CI 1.83–7.68) [47]. Despite this established association, obesity remains a relatively underappreciated VTE risk factor in long-term VTE management, highlighting the need for greater consideration in clinical practice.

### 3.4. New Considerations

In addition to traditional VTE risk factors, emerging influences are increasingly recognised as important contributors to VTE epidemiology. With increasing awareness and acceptance of gender diversity, attention has turned to the impact of gender affirming hormonal therapy (GAHT) on VTE risk in the transgender population. A recent meta-analysis found a two-fold increase in VTE risk in transgender women compared to both cisgender men and women (OR 2.23; 95% CI 1.93–2.57, *p* < 0.001) [9]. Conversely, there was no difference in risk in transgender men compared to ciswomen. In contrast to conventional oestrogen therapy, the VTE risk appears to increase with the duration of GAHT [48]. The optimal management and risk stratification of this cohort, however, remains uncertain.

Outside of individual clinical factors, social and environmental determinants also influence VTE risk. Lower socioeconomic status (SES) has been found to be an independent risk factor for VTE. Kort et al. demonstrated higher SES neighbourhoods had lower VTE incidence, though were unable to adjust for confounding factors such as pregnancy or obesity [49]. A later Danish study yielded similar findings, with risk persisting after adjusting for confounders, including obesity, cancer, cardiovascular disease and recent surgery (OR 0.61 vs. low SES score; 95% CI 0.59–0.63) [50]. This would indicate that VTE risk may be influenced by social determinants of health, beyond traditional clinical risk factors. Environmental exposures may further modify risk. Air pollution has been recognised as a risk factor for cardiovascular disease and more recently, there is emerging literature suggesting an association to VTE, through prothrombotic and proinflammatory pathways [51]. The Multi-Ethnic Study of Atherosclerosis (MESA) reported increased risk of VTE (HR 1.39; 95% CI 1.04–1.86) with higher average of air pollution indices [52]. However, Woller et al. found little evidence for short-term air pollution [53]. While the current literature examining the SES and environmental effect is scarce, it highlights the need to consider socioenvironmental factors when assessing risk and management of VTE.

Together, the evolution of known VTE risk factors and emergence of novel considerations highlight the complexity and heterogeneity of the at-risk population. Consequently, standard anticoagulant therapy is unlikely to be appropriate for all patients. Current risk stratification models, derived from historical cohorts, now likely underestimate risk profiles, underscoring the need to modernise these assessments to more accurately assess the contemporary population.

## 4. Treatment—Present Approaches and Future Considerations

### 4.1. The DOAC Era—Current Practices and Unresolved Issues

The introduction of DOACs over a decade ago transformed the treatment of VTE. Now the mainstream treatment, DOACs offers a number of advantages over vitamin K antagonists, including the ease of administration and more predictable pharmacokinetics, fewer drug interactions and no requirements for routine monitoring [54]. Landmark trials have consistently demonstrated non-inferior efficacy and reduced rates of bleeding compared with warfarin [10,11,12,13,14]. Real-world data continues to support the benefit of DOACs, with large retrospective cohort studies showing consistent reduction in recurrent VTE without increased bleeding, including patients excluded from initial trials, such as those with active cancer, antecedent surgery or trauma [55,56]. Similar findings have been shown in extended treatment and prophylaxis with DOACs. In the cohort study conducted by Fang et al., patients treated beyond 6 months with DOAC were found to have lower rates of recurrence (adjusted HR 0.66; 95% CI 0.52–0.82) with similar bleeding rates to warfarin (adjusted HR 0.79; 95% CI 0.54–1.17) [57]. Similar findings were demonstrated in the analysis by Lui et al., where additional sub-analysis of first unprovoked VTE identified significantly reduced recurrence (HR 0.30, 95% CI: 0.10–0.90, *p* = 0.03) but similar bleeding rates (HR 0.53, 95% CI 0.22–1.28, *p* = 0.158) on DOAC prophylaxis [58].

While DOACs are now widely recommended and utilised in patient cohorts initially excluded from trials, such as cancer-associated VTE [59] and obesity [60], caveats and evidence gaps remain. VTE prophylaxis is often underused in high-risk cancer patients, particularly those with gastrointestinal or genitourinary cancers, thrombocytopenia or renal impairment. Risk assessment tools such as the Khorana score have limited precision in contemporary oncology, as they do not account for newer therapies like ICIs or incorporate modern biomarkers. Clinicians therefore must balance evolving therapy-related thrombotic risks against bleeding risks, leading to inconsistent use of prophylaxis in practice.

The same fundamental challenge of balancing bleeding and thrombotic risk exists for therapeutic anticoagulation, especially with respect to optimal treatment duration. Current guidelines recommend long-term anticoagulation after a first unprovoked VTE in patients with low bleeding risk, largely based on a reported recurrence rate of 30% within five years of the event [61,62,63]. Comparatively, indefinite anticoagulation for unprovoked VTE was approached more cautiously during the warfarin era, due to bleeding risk and burden of therapy leading to treatment cessation [64]. This remains an area of clinical equipoise: up to 70% of patients receiving anticoagulation may not have a further event, exposing low-risk individuals to bleeding risk, up to approximately 1–2% [65]. One meta-analysis demonstrated increased clinically relevant non-major bleeding with extended anticoagulation [66]. This highlights the need for careful selection of patients who genuinely benefit from long-term anticoagulation.

Importantly, this uncertainty is not limited to unprovoked VTE. For provoked VTE, international guideline consensus suggests a limited course of anticoagulation for 3 months [61,67]. However, in the recently published HI-PRO study, patients with provoked VTE treated with prophylactic apixaban for 12 months had reduced VTE recurrence compared to those who received a limited 3-month course of anticoagulation (HR 2.68, 95% CI 0.96–7.43, *p* = 0.06) [68]. Notably, several provoking factors that were included could be considered minimally provoking, such as blood transfusions and exacerbation of chronic medical conditions. Furthermore, patients included in this study had at least one persistent VTE risk factor, such as chronic disease and obesity. While it may be debatable whether all cases were truly transiently provoked, the study highlights the heterogeneous definitions of provoking factors, as well as the need to consider the contribution from other non-traditional factors. The heterogeneity and variable potency of provoking factors, grouped under broad categories in current guidelines, leads to oversimplification. For example, recurrence after travel-provoked VTE appears similar to unprovoked VTE, suggesting that some transiently provoked events may reflect underlying thrombotic predisposition [69]. Together, these observations highlight that current guidelines may not fully capture the real-world complexities of “provoked” VTE, underscoring the urgent need for more nuanced risk stratification strategies.

Another area of uncertainty concerns patients with obesity. While ISTH guidance supports rivaroxaban and apixaban for obese patients regardless of body weight and BMI [60], there was significant heterogeneity and paucity of data at extremes of weight (BMI ≥ 50 kg/m^2^ and weight > 150 kg), as most studies classified “high weight” as BMI ≥ 30 kg/m^2^ and weight > 120 kg. Pharmacokinetic studies suggest altered drug exposure with increasing weight for apixaban and dabigatran, with little impact on rivaroxaban [70,71,72]. However, the clinical significance of this is unclear, and no DOAC has a clinically validated therapeutic range. Evidence is particularly limited for high-risk groups, such as patients after bariatric surgery, where available data are restricted to small studies and case reports with mixed findings [73,74]. Importantly, there is a lack of prospective randomised trials and long-term data to evaluate DOAC performance in patients with extremes of weight. Taken together, these limitations support cautious interpretation of current recommendations and underscore the need for well-designed trials for under-studied, high-risk populations.

### 4.2. Beyond DOACs–Novel Factor Inhibitors

Although DOACs have similar or lower bleeding risk compared to warfarin for VTE treatment, the annual bleeding rate of approximately 1% remains concerning. This apprehension is particularly evident in high-risk cohorts, such as advanced renal disease, where there are discordant opinions between regulatory bodies as to what is considered “safe”. The U.S. Food and Drug Administration (FDA) has approved use of apixaban in end-stage renal failure and haemodialysis based on pharmacokinetic data, whereas the European Medicines Agency is more conservative, recommending against its use in this cohort [75,76]. Compounding this concern, reversal agents for DOACs are limited. Andexanet alfa, a recently introduced reversal agent for factor Xa inhibitors, is not readily available, expensive, and associated with thrombotic risk of up to 10% [77]. Notably, andexanet alfa was withdrawn from the United States market in late 2025, following FDA concerns that thromboembolic risks outweigh benefits [78]. Its future use remains uncertain, though similar recommendations have yet to be made worldwide. As such, these limitations highlight the need for safer anticoagulation strategies and more accessible, effective reversal agents in high bleeding risk patients.

FXI has emerged as a target for anticoagulant therapy, based on observations in congenital FXI deficiency patients, who rarely experiencing spontaneous bleeding and have reduced VTE incidence [79,80,81]. Several FXI inhibitors are currently in early phase clinical trials. In haemodialysis patients, early signals would suggest no increased bleeding risk, though larger studies are required to assess their efficacy [82]. FXI inhibitors have also been explored as VTE prophylaxis for patients undergoing total knee replacement, with phase II trials demonstrating non-inferior, if not superior, reduction in VTE compared to enoxaparin [83]. However, larger trials have produced mixed signals. The Phase III ASTER (NCT05171049) trial comparing abelacimab to apixaban in CAT was terminated early after interim analysis, though results have not yet been formally released [84]. This decision was also extended to the MAGNOLIA (NCT05171075) trial, which compared abelacimab to dalteparin for CAT in gastrointestinal and genitourinary cancers, due to strategic and operational considerations [85]. In contrast, the AZALEA-TIMI 71 trial conducted in patients with moderate-to-high risk of stroke was stopped early due to a greater than anticipated reduction in bleeding events with abelacimab [86]. A summary of FXI inhibitor trials for VTE prevention and treatment is shown in Table 1. FXII and factor XIII (FXIII) have also been examined as potential therapeutic targets. FXII inhibitors have largely been investigated in murine models, demonstrating reduced thrombotic events, while FXIII inhibitors have yet to enter clinical development [87].

Overall, the current evidence base for these novel inhibitors remains early and largely focused on prophylaxis. Their efficacy and safety for treatment of VTE remain uncertain. Real-world data are absent, and long-term safety has not been established. If these novel agents prove safe and effective in high-risk patients with multimorbidity, including advanced CKD, they could represent a major advance. They would provide a much-needed therapeutic option in patients where DOACs are limited by renal clearance and heightened bleeding risk.

### 4.3. Endovascular Approaches

While anticoagulation remains the mainstay of treatment for VTE, an important limitation is the inability to rapidly remove existing thrombus. Anticoagulants prevent further clot propagation but do not acutely reduce clot burden, and may not avert complications such as haemodynamic instability in PE or post-thrombotic syndrome (PTS) in iliofemoral DVT. These limitations have driven growing interest in interventional therapies in select patient populations where rapid clot debulking may improve clinical outcomes.

For PE, current guidelines suggest anticoagulant therapy is adequate for low-risk patients, while systemic thrombolysis is indicated for unstable high-risk patients [88]. In contrast, the optimal management of intermediate-risk PE remains unclear. Mortality risk associated with this subgroup has been reported to be as high as 3–15%, with up to 38% risk of deterioration [89,90]. Although systemic thrombolysis can reduce early haemodynamic compromise, the substantial risk of major bleeding, particularly intracranial haemorrhage, precludes its routine use in intermediate-high risk PE [91,92]. This highlights the need to refine risk stratification to better identify patients at highest risk of deterioration who may benefit from additional intervention, without concurrent excessive treatment-related harm.

Large-bore mechanical thrombectomy (LBMT), a minimally invasive endovascular procedure that allows aspiration of thrombus without thrombolytics, has emerged as a potential therapy for intermediate-risk PE. The benefit of LBMT is derived from rapid haemodynamic improvement, in part due to resolution of pulmonary hypertension and right heart strain [93]. In the PEERLESS trial, conducted predominantly in intermediate-high risk patients, LBMT was superior to catheter-directed thrombolysis (CDT) with a lower composite primary end point (win ratio 5.01; 95% CI 3.68–6.97, *p* < 0.001) and significantly lower rates of clinical deterioration, need for bailout therapy, ICU utilisation and length of stay [17]. Rates of intracranial and major bleeding were comparable between the groups, though high bleeding risk patients were excluded, likely underestimating complications. Similarly, the recent STORM-PE trial evaluated computer-assisted vacuum thrombectomy with anticoagulation versus anticoagulation alone in intermediate-high risk PE, reporting superior reduction in RV/LV ratio with comparable major adverse events within 7 days [94]. Building on PEERLESS, the active PEERLESS II trial is comparing LBMT to anticoagulation alone in the intermediate risk cohort [95], while the HI-PEITHO and PE-TRACT studies are concurrently investigating CDT in intermediate-risk PE [96,97]. These studies may redefine the optimal management of intermediate-risk PE, though current evidence is limited by selective enrolment and short-term surrogate outcomes, with lack of data on chronic thromboembolic pulmonary hypertension. Consequently, the extent to which endovascular interventions improve clinically meaningful outcomes, and how they should be integrated into routine care for intermediate-risk PE, remains uncertain. At present, their applicability appears limited to intermediate-high risk patients with haemodynamic or right ventricular compromise, risk of further deterioration or failure of anticoagulation.

Interventional approaches for DVT have also been controversial, with mixed efficacy and safety findings. Management of iliofemoral DVT has been of particular interest, with up to 50% of patients developing PTS [98], and nearly a twofold risk compared to those without iliac vein involvement when managed with anticoagulation alone [99]. The CaVenT trial was one of the first major trials to examine the efficacy of CDT in addition to anticoagulation in iliofemoral DVT. At 6 months follow up, iliofemoral patency was improved compared to anticoagulation alone, with absolute risk reduction (ARR) of 28.2% (95% CI 9.7–46.7; *p* = 0.004) [99]. However, despite a significant reduction in PTS at 5 years, there was no improvement in quality of life [100]. Subsequent studies have demonstrated mixed results; findings are summarised in Table 2. In the ATTRACT trial, while rates of moderate/severe PTS in the iliofemoral cohort were reduced, the overall PTS rate was not, and more major bleeding events were observed within 10 days [101]. Similarly, the CAVA trial reported no reduction in PTS, with more recurrent DVT events due to in-stent thrombosis in the intervention arm [102]. Long-term follow-up, however, showed reduction in PTS, albeit using ISTH PTS criteria as compared to Villalta score in the original study, with significant reduction only in mild PTS [103].

Significant heterogeneity exists between the three trials due to clinical and methodological differences, which likely explains the variability across the studies. For instance, the inclusion of femoropopliteal DVT in the ATTRACT study may have diluted treatment effects. Sub-analyses found reduced PTS severity for iliofemoral DVT, whereas there was no benefit for femoropopliteal DVT [104,105]. Timing of intervention also varied; earlier treatment is thought to improve the chance of successful thrombus removal before chronic venous obstruction occurs. Additionally, different interventional modalities were used. CaVenT and ATTRACT used pharmacological CDT with anticoagulation, whereas CAVA used ultrasound-assisted CDT with adjuncts at clinician’s discretion, introducing heterogeneity in efficacy and complication rates. Finally, disparate endpoints and definitions of PTS further complicate interpretation. Given these inconsistences and the observed risk of major bleeding, current data only support a limited role for CDT in patients with iliofemoral DVT who are low bleeding risk.

In addition to CDT, LBMT has shown early promise in the treatment of iliofemoral DVT. Interim analysis of the CLOUT registry demonstrated significant improvement in median Villalta score, from 9 (interquartile range, IQR 5–14) to 1 (IQR 0–3; *p* < 0.001) at 6-month analysis [106]. This improvement persisted at 1-year follow up [107]. Notably, this cohort included iliofemoral and femoropopliteal DVT, with any symptom duration, though half the patients had less than seven days of symptoms. No major bleeding events were reported, noting only a third of patients had a contraindication to thrombolytic therapy. In comparison to the ATTRACT trial, patients treated with LBMT had reduced 12-month incidence of PTS after propensity score matching (17% vs. 38%; *p* < 0.001) [108]. While further experience is required, LBMT may offer an attractive interventional option in higher bleeding risk patients, with the upcoming DEFIANCE study aiming to compare LBMT with anticoagulation for iliofemoral DVT [109].

Taken together, these findings indicate that endovascular approaches for DVT should be applied selectively. The most consistent evidence of benefit appears in patients with acute (<14 days), symptomatic iliofemoral DVT and low bleeding risk, especially those with significant symptom burden or threatened venous outflow. Outside of these scenarios, the data remains inconsistent and routine use is not recommended. Procedural variability, inconsistent patient selection and heterogeneous endpoints highlight the need for better designed trials. Until such evidence emerges, endovascular interventions should be reserved for carefully selected individuals following multidisciplinary discussion.

### 4.4. Outpatient Management Models

While VTE management has traditionally occurred in a hospital setting, the availability of oral anticoagulants, and more recently DOACs, has allowed for the safe management of many patients in the ambulatory setting. Outpatient management has been of particular interest in low-risk PE, with several international guidelines now recommending that appropriately risk-stratified patients can be managed in the community [67,88,110]. However, the uptake of this management strategy has been limited, with no consensus criteria. In the HOME-PE trial, 2000 patients were randomised to triaging by either Hestia criteria or sPESI [19]. There was no significant difference in the composite primary outcome of recurrent VTE, major bleeding or all-cause death within 30 days (*p* = 0.004 for non-inferiority). Importantly, a sPESI score of 0 was overruled in nearly 30% of patients compared to only 3% with a negative Hestia score. This highlights the importance of other considerations, such as psychosocial factors.

Comparable successes have been achieved in other smaller studies, including the MATH-VTE, LOPE and HoT-PE trials, which utilised additional clinical criteria with or without validated prognostic scores to characterise low-risk patients [111,112,113]. Fresh consideration has focused on establishing consensus criteria, while addressing the limitation of current prognostic scores. The recently published EARTH (Emergency Advisory and Research international board on Thrombosis and Hemostasis) rule incorporates consideration of social factors, bleeding risk and assessment of right ventricular dysfunction to address the deficiencies in the PESI, sPESI and Hestia criteria [114]. Pending validation, this criterion may help guide the appropriate selection of patients for outpatient management.

DOACs have reshaped VTE management, but important uncertainties remain for complex patients, while interventional approaches can be considered in selected patients. Emerging factor XI inhibitors may help reduce bleeding, although more evidence is required. Box 1 summarises some of the key treatment considerations.

Box 1 **Treatment key messages.**

- While DOACs have transformed VTE management, uncertainty remains regarding the optimal treatment of complex patient cohorts, highlighting the limitations of current guidelines and the need for nuanced risk stratification.
- Novel FXI inhibitors aim to mitigate the limitations of anticoagulation in high bleeding risk cohorts; however, evidence remains early and largely untested in real-world settings.
- Interventional approaches can enable rapid thrombus removal, but current evidence only supports use in carefully selected intermediate-high risk PE and iliofemoral DVT patients.
- Outpatient management of low-risk PE is feasible, though its practice remains limited.

## 5. Risk Stratification—Current Tools and Emerging Needs

### 5.1. Current Limitations

Risk assessment models (RAMs) are helpful in guiding management of VTE, though opportunities remain to improve precision and clinical applicability, as well as develop novel tools. Clinical acumen and patient-reported symptoms commonly feature in RAMs, such as the Wells criteria for DVT and PE, or the Villalta score for assessing PTS severity [115,116,117]. While inter-operator reliability for these tools is generally high, subjectivity remains and potentially affects reproducibility [118,119]. That is not to say clinical features are unhelpful in risk assessment; rather, they provide valuable context that is otherwise unassessable with diagnostic tests or other objective measures. This is emphasized with the Hestia criteria, where consideration is given to psychosocial factors that prevent safe outpatient management of patients with otherwise low-risk PE [120]. Furthermore, many RAMs used in current clinical practice only cover specific patient cohorts. For example, the Khorana score, used in assessment of VTE risk in cancer patients, performs poorly in certain cancer subtypes [121]. It also does not capture the contribution of cancer therapies to risk. For the risk of recurrence in unprovoked VTE, the HERDOO2, Vienna and DASH scores are largely unvalidated in men, cancer patients and those with high-risk thrombophilia [122]. These gaps in validated RAMs highlight an opportunity to develop novel risk stratification tools that can improve accuracy and applicability to a diverse cohort of patients.

Currently, most widely used RAMs lack integration of reliable biomarkers. In contrast to clinical criteria, biomarkers offer a more objective assessment of patients’ haemostatic profile. D-dimer is the most commonly used biomarker, incorporated into the Wells criteria and PERC scores to guide the need for imaging for DVT or PE. However, D-dimer is highly non-specific, elevated by inflammation as well as age, with no consensus for thresholds and considerable assay variability [123,124]. In the same vein, the Khorana score utilises haematological parameters, which are surrogate markers but lack specificity for thrombosis. Additionally, while able to identify high-risk patients, the incidence of VTE has been shown to be considerably elevated in lower risk groups, limiting its clinical utility [121]. However, when combined with D-dimer and p-selectin in the Vienna CATS score, risk stratification is enhanced compared to the original Khorana score [125]. Thus, identification and incorporation of relevant biomarkers into RAMs will likely enhance their predictive power.

### 5.2. Biomarkers and Novel Assays

Biomarkers have been increasingly explored to further improve VTE risk stratification. Notably, elevated factor VIII (FVIII) has been associated with VTE risk. The landmark Leiden Thrombophilia Study demonstrated FVIII activity levels > 150 IU/dL were associated with an approximately 5-fold increase in VTE (OR 4.8, 95% CI 2.3–10.0) [126]. In a meta-analysis by Teymoori et al. examining 18 studies, FVIII activity levels > 200 IU/dL was associated with increased VTE recurrence (RR = 1.70, 95% CI 1.32–2.19; *p* < 0.001) [127]. Notably there was significant heterogeneity between studies, limiting interpretability. Another systematic review found mixed association of FVIII to VTE, again with significant heterogeneity, preventing meta-analysis [128]. In comparison to p-selectin, FVIII assays are much more widely available; however, they have yet to be integrated into any RAMs. Furthermore, as FVIII levels are affected by factors such as age, inflammation and timing from the acute event, careful consideration of threshold values will be necessary before incorporation into RAMs [128,129,130].

P-selectin has also emerged as one of the major novel biomarkers in predicting VTE. It is a transmembrane protein stored in platelet α-granules and endothelial Weibel-Palade bodies and released upon cell activation, leading to cell surface expression and release of soluble p-selectin into plasma [131,132]. Soluble p-selectin has been shown to be significantly elevated in patients with VTE, even with concurrent medical comorbidities, including solid organ malignancy, HIV and lupus anticoagulant [133]. It has also been associated with recurrent VTE in patients with prior unprovoked VTE [134], as well as development and recurrence of CAT in both solid organ and haematological malignancy [135,136]. A recent prospective study in the Tromsø population evaluated soluble p-selectin in predicting VTE in patients with no prior VTE [137]. The authors discovered that elevated soluble p-selectin levels in the highest quartile were associated with increased VTE risk compared to the lowest quartile in women (OR 1.63; 95% CI 1.01–2.64). Paradoxically, higher levels of soluble p-selectin were associated with lower VTE risk in men (OR 0.69; 95% CI 0.42–1.15), the pathophysiology of which is unknown. This sex specific difference will need to be addressed before p-selectin can be more broadly incorporated into RAMs. Nonetheless, current studies, as well as the aforementioned Vienna CATS score, demonstrate the potential additive value of p-selectin in VTE risk assessment.

Tissue-factor bearing microparticles (TF-MP) have been previously shown to expressed at higher levels in cancer patients compared to healthy controls [138]. Increased TF-MP activity has also been seen in cancer patients with VTE compared to those without [139]. These findings were agreed upon in a meta-analysis conducted by Cui et al., where TF-MP have been shown to be associated with VTE risk in cancer patients (OR = 1.76, 95% CI 1.21–2.56) [140]. The association appears to be conflicting in the general population. Ay et al. found no association between TF-MP and VTE risk in their age and gender matched cohort [141]. Conversely, Bal et al. demonstrated acutely elevated levels in PE patients, while Ye et al. demonstrated the same in patients with recurrent DVT [142,143]. However, TF-MP can be elevated in a number of different diseases in addition to cancer, such as liver and cardiovascular disease, as well as active infections [144]. These factors will need to be accounted for before TF-MP are used more widely in VTE risk stratification.

While individual biomarkers provide insight into components of the coagulation pathway and the pathophysiology of thrombosis, they offer only a limited snapshot of the coagulation cascade. Global coagulation assays, which capture the interplay of pro- and anti-coagulant factors, provide a more comprehensive assessment of thrombotic potential and may have a role in VTE risk stratification. The Calibrated Automated Thrombogram (CAT), an assay that assesses thrombin generation, has been the most widely studied in this area. These assays have previously been shown to be independently associated with VTE recurrence [145,146]. There appears to be significant variability in methods, with other studies demonstrating association with risk of first VTE but not recurrence, though both had methodological issues in sample preparation and timing of the assays [147,148]. Conversely, The Overall Haemostatic Potential (OHP) assay simultaneously assesses fibrin formation and fibrinolysis in a single test system [149]. In small pilot studies, OHP has demonstrated promise as a risk stratification tool for VTE recurrence and PTS [150,151].

In addition to the biomarker specific limitations, there are several overarching barriers that limit the wider integration of novel biomarkers and global coagulation assays in RAMs. Besides FVIII, most exist primarily as research tools, with no standardised protocols, established reference method, validated cut-offs or clinical decision points. Furthermore, many of these assays are sensitive to pre-analytical variables such as sample timing, acute inflammation, and patient demographics and comorbidities. For instance, technical factors such as a difficult blood draw or even needle gauge may affect the outcomes of TF-MP results [152]. Inter-lot variability is also common in research-based assays and is another barrier to reproducibility. This was illustrated by Feng et al., where an average of 62.4% lot-to-lot variability was identified in their biomarker control assays [153]. These uncertainties undermine their reliability as standalone predictors. Importantly, very few studies have evaluated how these biomarkers perform across diverse, real-world populations and demonstrated meaningfully improve risk prediction beyond established clinical factors. Larger, prospective validation studies, harmonised assay methodology and clarification of threshold values are essential before these biomarkers can be integrated into routine VTE RAMs or guide clinical decision making.

### 5.3. The Role of Artificial Intelligence

Another key limitation of current VTE RAMs is the largely static combinations of clinical and laboratory variables, which may not fully represent an individual’s dynamic VTE risk. Artificial intelligence (AI) has emerged as a tool in medicine with potential to improve diagnosis and risk stratification, and has been explored in VTE for primary prevention and diagnosis [154]. In particular, machine learning (ML) models are able to adapt to data over time and adjust their risk assessments accordingly. In one pooled analysis of 20 studies, predominantly ML models, these methods demonstrated superior ability to predict first VTE events (area under curve, AUC 0.79 vs. 0.61 for conventional models) [155]. Another meta-analysis examining 27 ML based models reported strong predictive ability for VTE (c-index 0.84; 95% CI 0.8–0.88) [156]. However, both analyses, despite demonstrating predictive ability, highlight several key shortcomings. Many included models were constructed from niche or small populations and failed to provide details regarding inclusion and exclusion criteria. Study design and intended use cases of models were heterogenous, limiting direct comparisons between models. High risk of bias was also observed, due to lack of details on eligibility criteria, model calibration, outcome definitions and handling of missing data. Furthermore, only 2 (10%) and 4 (15%) of studies in the respective reviews were externally validated, raising the possibility that reported performance is overestimated. Together, these issues, along with the limited evidence base, temper the current clinical utility of ML models. Careful validation in diverse clinical settings will be vital before routine use.

Similar to the lack of gold-standard assays for novel biomarkers, there is no single “benchmark model” for AI models. For example, in one study examining ML models for the prediction of VTE in patients with coronary artery disease, the extreme gradient boosting (XGBoost) model performed the best in internal validation [157]. However, other models had higher AUC and specificity on external validation. In hospitalised cancer patients, XGBoost was found to be the best predictive VTE model [158], yet performed worse than the random forest model when examining postoperative VTE risk in cervical cancer patients [159]. Different clinical scenarios may favour different ML models, representing another complexity in implementation.

Beyond the need for further development and validation, the implementation of AI in healthcare settings raises concerns regarding the transparency, automation bias, deterioration of clinical acumen and ethical and legal implications [160]. Surveys of informaticians and clinicians reflect these concerns, with agreement that while AI models can assist in VTE management, clinical judgement remains paramount [161]. Successful adoption of ML models for VTE risk assessment depends not only on the technical aspects of design and implementation, but also on clinician acceptance and engagement. Large, prospective, real-world studies, with transparent reporting and validation across multiple settings, are essential before these tools can be integrated into routine clinical practice.

Current risk assessment models have limited individual level accuracy while emerging biomarkers and artificial intelligence still require validation in real-world settings. Box 2 summarises some of the considerations for current and future risk stratification strategies.

Box 2 **Risk stratification key messages.**

- Current RAMs are based on historical cohorts with minimal biomarker integration and may not accurately assess risk in contemporary populations.
- Novel biomarkers, such as FVIII, p-selectin and TF-MP, as well as global coagulation assays may provide more objective assessments of thrombotic risk, but require standardisation and real-world validation.
- AI and ML models represent another tool to improve VTE risk stratification, but face technical, ethical and clinician acceptance barriers.

## 6. Conclusions

**Figure 1:**
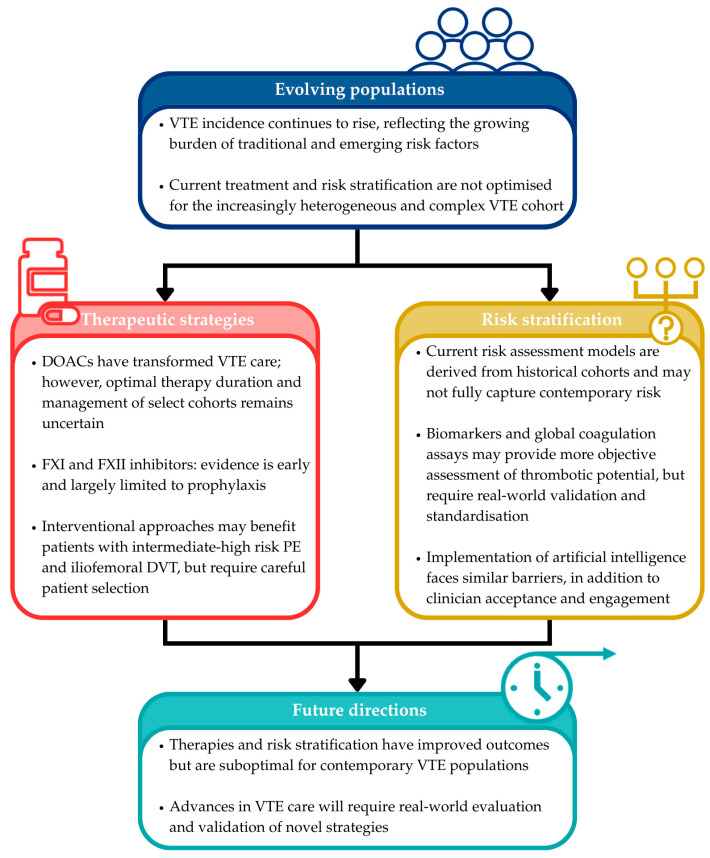
Conceptual summary based on the published literature.

The contemporary VTE patient cohort is increasingly heterogeneous and complex, driven by rising traditional risk factors and emerging contributors. This shift poses new challenges for treatment and risk stratification. While DOACs have transformed care by offering simpler, safer and effective anticoagulation, important gaps remain for patients with high bleeding risk and optimal management for select cohorts remains unclear. Novel FXII and FXIII inhibitors and interventional strategies for PE and DVT show early promise but remain under investigation, and may only benefit select patients. Concurrently, current risk assessment models are poorly aligned with modern VTE populations. While novel biomarkers offer objective insight into thrombotic potential, their use is limited by lack of standardization and clinical validation. The use of AI and ML models may offer an alternate risk stratification strategy, but face major barriers including limited external validation and methodological heterogeneity for now. Collectively, these innovations, summarised in Figure 1, hold potential to reshape VTE management. However, widespread adoption will require rigorous evaluation, standardisation and validation to ensure meaningful improvements in real-world outcomes.

## Figures and Tables

**Table 1:** Summary of FXI inhibitor trials in VTE treatment and prophylaxis, adapted from Steiner et al. [82] and Gailani and Gruber [83].

Clinical Trial (Drug)	Cohort and Indication	Control	Thromboembolism Incidence
FXI-ASO TKA (Ionis FXI_Rx_)	TKA, prophylaxis	Enoxaparin	Non-inferior (200 mg) Superior (300 mg) *
FOXTROT (Osocimab)	TKA, prophylaxis	Enoxaparin Apixaban	Non-inferior ^†^ (post-operative 0.6, 1.2, 1.8 mg/kg) Superior ^†^ (pre-operative 1.8 mg/kg) *
ANT-005 TKA (Abelacimab)	TKA, prophylaxis	Enoxaparin	Non-inferior (30 mg) Superior (75 mg, 150 mg) *
AXIOMATIC-TKR (Milvexian)	TKA, prophylaxis	Enoxaparin	Superior (200 mg daily; 50 mg, 100 mg, 200 mg bd) *
Lorentz et al. (Gruticibart)	HD, prophylaxis	Placebo	– ^§^
CS4 (Ionis FXI_Rx_)	HD, prophylaxis	Placebo	– ^§^
CONVERT (Osocimab)	HD, prophylaxis	Placebo	No significant difference ^¶^
RE-THINC (Fesomersen)	HD, prophylaxis	Placebo	No significant difference ^¶^
EMERALD (Ionis FXI_Rx_)	HD, prophylaxis	Placebo	– ^^^
ASTER (Abelacimab)	CAT, therapeutic	Apixaban	– ^^^
MAGNOLIA (Abelacimab)	CAT, therapeutic	Dalteparin	– ^^^

Incidence of thromboembolism as compared to control arm. There was no significant difference in bleeding compared to control arms in any study (excluding the ASTER and MAGNOLIA trials where data is not available). * Specifically for VTE incidence. ^†^ Only when compared to enoxaparin. ^§^ Thromboembolic events not reported. ^¶^ Pooled analysis of all evaluated doses. ^^^ Data insufficient or not published for analysis. Abbreviations: TKA—total knee arthroplasty, HD—haemodialysis, CAT—cancer associated thrombosis, bd–twice daily.

**Table 2:** Summary of key interventional trials for DVT, including long-term follow up.

Study	Cohort	Outcomes
CaVenT (CDT)	Iliofemoral DVT, including proximal femoral DVT Symptom onset ≤ 21 days	Reduced PTS at 24 months (ARR 14.4%, 95% CI 0.2–27.9; *p* = 0.047) Reduced PTS at 5 years (ARR 28%, 95% CI 14–42; *p* < 0.0001) 3 major bleeding events vs. 0 in control arm *
ATTRACT (CDT)	Iliofemoral and femoropopliteal DVT Symptom onset ≤ 14 days	Between 6–24 months Reduced moderate/severe PTS (RR 0.73, 95% CI 0.54–0.98; *p* = 0.04) No reduction in PTS overall (RR 0.96, 95% CI 0.82–1.11; *p* = 0.56) Increased major bleeding at 10 days (1.7% vs. 0.3%; *p* = 0.049)
CAVA (UA-CDT ± angioplasty/ stenting)	Iliofemoral DVT Symptom onset ≤ 14 days	No reduction in PTS at 12 months (OR 0.75, 95% CI 0.38–1.50; *p* = 0.42) Reduced PTS at 39 months ^†^ (OR 0.40, 95% CI 0.19–0.84; *p* = 0.01) 17 episodes of recurrent DVT (including 12 in-stent thromboses) vs. 7 episodes in control arm 4 major bleeding events vs. 0 in control arm (OR 9.25, 95% CI 0.49–174.7)
CLOUT registry (LBMT)	Iliofemoral and femoropopliteal DVT Any symptom duration	Improvement in PTS at 12 months Villalta score, 9 (IQR 5–14) to 1 (IQR 0–4; *p* < 0.001) No major bleeding events reported

* Statistical significance not reported. ^†^ Using ISTH PTS criteria. Abbreviations: CDT—catheter directed thrombolysis, UA-CDT—ultrasound accelerated catheter directed thrombolysis, ARR—absolute risk reduction, IQR—interquartile range.

## Data Availability

No new data were created or analyzed in this study. Data sharing is not applicable to this article.

## Supplementary Materials

The following supporting information can be downloaded at: https://www.mdpi.com/article/10.3390/jcm15041509/s1, Table S1: Database search strategy.
