# Supplementary material for: Contemporary Challenges in Venous Thromboembolism: Evolving Populations and Implications for Management and Risk Stratification
Source: J Clin Med. 2026 Feb 14;15(4):1509. doi: 10.3390/jcm15041509 (PMC12942584; doi:10.3390/jcm15041509)
Supplement: Supplementary file 1 [file jcm-15-01509-s001.zip › jcm-4158114-supplementary.pdf]

Supplementary Table S1. Database search strategy

**SEARCH TERMS**<br>Core<br>("Venous Thromboembolism"[MeSH Terms] OR "deep vein thrombosis"[MeSH Terms] OR "pulmonary embolism"[MeSH Terms] OR "venous thromboembolism"[tiab] OR VTE[tiab] OR "deep vein thrombosis"[tiab] OR DVT[tiab] OR "pulmonary embolism"[tiab] OR PE[tiab])<br><br>Epidemiology and modifiers<br>("Epidemiology"[MeSH] OR "Incidence"[MeSH] OR "Prevalence"[MeSH] OR "Risk Factors"[MeSH] OR "Mortality"[MeSH] OR epidemiolog* OR incidence OR prevalence OR risk factor* OR trend* OR burden OR lifetime risk OR cohort OR population-based OR population based)<br>("Neoplasms"[MeSH] OR cancer* OR malignan* OR tumor* OR tumour* OR immunotherapy OR CAR-T)<br>("Obesity"[MeSH] OR "Body Mass Index"[MeSH] OR obesity OR BMI OR body mass index OR leptin)<br>("Transgender Persons"[MeSH] OR transgender* OR gender-affirming OR feminizing hormone therapy)<br>("Socioeconomic Factors"[MeSH] OR socioeconomic* OR deprivation OR income OR education)<br>("Air Pollution"[MeSH] OR "Particulate Matter"[MeSH] OR air pollution OR PM2.5 OR ozone)<br><br>Treatment<br>*DOACs*<br>(treatment[tiab] OR therapy[tiab] OR management[tiab] OR anticoagulant therapy[tiab] OR anticoagulation[tiab] OR "extended treatment"[tiab] OR "extended therapy"[tiab] OR "long-term"[tiab] OR "extended duration"[tiab])<br>AND<br>("anticoagulants"[MeSH Terms] OR "direct oral anticoagulants"[tiab] OR DOAC[tiab] OR "non-vitamin K oral anticoagulant"[tiab] OR NOAC[tiab] OR apixaban[tiab] OR rivaroxaban[tiab] OR dabigatran[tiab] OR edoxaban[tiab])<br><br>*Novel factor and interventional therapies*<br>("Factor XI"[MeSH Terms] OR "factor XI"[tiab] OR "factor XIa"[tiab] OR "factor XI inhibitors"[tiab] OR "factor XIa inhibitors"[tiab] OR abelacimab[tiab] OR osocimab[tiab] OR milvexian[tiab] OR asundexian[tiab] OR fesomersen[tiab] OR "FXI inhibitor"[tiab] OR "FXIa inhibitor"[tiab] OR "factor XI deficiency"[tiab] OR "FXI deficiency"[tiab])<br><br>("catheter-directed thrombolysis"[MeSH Terms] OR "thrombolytic therapy"[MeSH Terms] OR "thrombectomy"[MeSH Terms] OR catheter-directed thrombolysis[tiab] OR CDT[tiab] OR catheter-directed therapy[tiab] OR catheter directed therapy[tiab] OR mechanical thrombectomy[tiab] OR percutaneous thrombectomy[tiab] OR ultrasound-accelerated thrombolysis[tiab] OR ultrasound facilitated thrombolysis[tiab] OR pharmacomechanical thrombolysis[tiab] OR pharmacomechanical catheter-directed thrombolysis[tiab] OR endovascular thrombus removal[tiab] OR endovascular thrombus extraction[tiab]OR PEITHO[tiab] OR ATTRACT[tiab] OR CAVA[tiab] OR CLOUT[tiab])<br><br>*Outpatient management*

("outpatient"[MeSH Terms] OR "home care services"[MeSH Terms] OR outpatient[tiab] OR home treatment[tiab] OR home care[tiab] OR early discharge[tiab]OR ambulatory[tiab] OR outpatient management[tiab] OR "emergency department"[tiab] OR ED[tiab] OR "early discharge" OR "early discharge program"[tiab]OR "low-risk pulmonary embolism"[tiab] OR "low risk pulmonary embolism"[tiab] OR "home treatment of pulmonary embolism"[tiab])<br><br><u>Risk stratification</u><br>("Risk Assessment"[MeSH] OR "Prognosis"[MeSH] OR "Recurrence"[MeSH] OR "Decision Support Techniques"[MeSH] OR "Biomarkers"[MeSH] OR prognos*[tiab] OR predict*[tiab] OR risk stratification[tiab] OR clinical score*[tiab] OR prediction model*[tiab] OR outpatient*[tiab] OR ambulatory[tiab] OR recurrence[tiab] OR D-dimer[tiab] OR "P-selectin"[tiab] OR "factor VIII"[tiab] OR thrombin generation[tiab] OR endogenous thrombin potential[tiab] OR microparticle*[tiab] OR "tissue factor"[tiab])<br><br><u>Artificial intelligence</u><br>("Artificial Intelligence"[MeSH] OR "Machine Learning"[MeSH] OR "Algorithms"[MeSH] OR artificial intelligence OR machine learning OR deep learning OR algorithm*)<br>AND<br>("Risk Assessment"[MeSH] OR "Prognosis"[MeSH] OR "Models, Statistical"[MeSH] OR predict* OR prediction OR risk stratification OR risk assessment)
**INCLUSION STRATEGY**<br>• Studies reporting:<br>o Epidemiology: incidence, prevalence and trends in venous thromboembolism and associated risk factors.<br>o Therapy: anticoagulants, novel agents, interventional approaches, care pathways<br>o Risk stratification: existing clinical risk models, novel biomarkers (eg. TF-MP, p-selectin, FVIII, global coagulation assays), artificial intelligence.<br>• Observational studies, clinical trials, reviews and meta-analyses involving human participants.<br>o Relevant in vitro and translational studies were also included.<br>• Articles published in English.
**EXCLUSION STRATEGY**<br>• Animal studies or papers targeting pediatric populations<br>• Studies focusing exclusively on arterial thromboembolism without venous thromboembolism data<br>• Case reports, editorials or non-peer-reviewed sources (excluding grey literature and when used for context).<br>• Non-English publications.
